# Potential impact of influenza vaccine roll-out on antibiotic use in Africa

**DOI:** 10.1093/jac/dky172

**Published:** 2018-05-09

**Authors:** Gwenan M Knight, Madeleine Clarkson, Thushan I de Silva

**Affiliations:** 1 Centre for Mathematical Modelling of Infectious Diseases, London School of Hygiene and Tropical Medicine, Keppel Street, London, UK; 2 National Institute for Health Research Health Protection Research Unit in Healthcare Associated Infections and Antimicrobial Resistance, Imperial College London, London, UK; 3 Vaccines and Immunity Theme, Medical Research Council Unit, The Gambia at the London School of Hygiene and Tropical Medicine, Banjul, The Gambia; 4 Centre of International Child Health, Section of Paediatrics, Department of Medicine, Imperial College London, St Mary’s Campus, London, UK; 5 The Florey Institute for Host-Pathogen Interactions and Department of Infection, Immunity and Cardiovascular Disease, University of Sheffield, Sheffield, UK

## Abstract

**Background:**

Influenza infections result in both inappropriate and appropriate antibiotic prescribing. There is a huge burden of both influenza and infections caused by antimicrobial-resistant pathogens in Africa. Influenza vaccines have the potential to reduce appropriate antibiotic use, through reduction of secondary bacterial infections, as well as to reduce levels of influenza misdiagnosed and treated as a bacterial infection (inappropriate).

**Objectives:**

To estimate potential reductions in antibiotic use that are achievable by introducing an influenza vaccine into various African settings.

**Methods:**

Influenza incidence was combined with population size, vaccine and health system characteristics.

**Results:**

We estimated that the direct impact of vaccination could avert more than 390 prescriptions per 100 000 population per year if a 50% efficacious influenza vaccine at 30% coverage was introduced to adults >65 years old in South Africa or children 2–5 years old in Senegal. Across Africa, purely through reducing the number of severe acute respiratory infections, the same vaccine characteristics could avert at least 24 000 antibiotic prescriptions per year if given to children <5 years old.

**Conclusions:**

The introduction of an influenza vaccine into multiple African settings could have a dramatic indirect impact on antibiotic usage. Our values are limited underestimates, capturing only the direct impact of vaccination in a few settings and risk groups. This is owing to the huge lack of epidemiological information on antibiotic use and influenza in Africa. However, it is likely that influenza vaccination in Africa could substantially impact antibiotic usage in addition to influenza-related mortality and morbidity.

## Introduction

Antimicrobial resistance (AMR) is a global concern. The rise in resistance, in part, is attributed to inappropriate use of antibiotics such as for misdiagnosed viral infections, including influenza. Currently, the capacity to tackle misdiagnosis is lacking in many low- and middle-income countries (LMICs). A recent review of AMR in Africa highlighted high levels of resistance to antibiotics commonly used for respiratory tract infections.^1^ Moreover, West and Southern Africa had among the greatest increases globally in per person antibiotic consumption between 2000 and 2010.^2^

Influenza infections result in increased antibiotic prescribing to treat secondary bacterial infections (appropriate) and primary influenza cases misdiagnosed as bacterial infections (inappropriate). An indirect benefit of influenza vaccination could be to reduce antimicrobial prescribing and, ultimately, AMR. However, both the burden of influenza and use of influenza vaccines in Africa have been neglected. A study of 15 African countries demonstrated that influenza accounted for 21.7% of influenza-like illness (ILI) and 10.1% of severe acute respiratory infection (SARI) cases.^3^ A recent systematic analysis found that the per capita influenza-associated hospitalization rate in children <5 years old was >3-fold higher in Africa compared with Europe.^4^

In 2012, the WHO Strategic Advisory Group of Experts recommended influenza vaccination in key high-risk groups: pregnant women (with potential protection for the neonate), children aged 6–59 months, the elderly, healthcare workers and those with specific chronic medical conditions. However, a recent analysis found that only three African countries (of 47 WHO member states) had implemented seasonal influenza vaccine policies.^5^

The Global Alliance for Vaccines and Immunization (GAVI) foundation, a major vaccine funder, has proposed immunization as a key strategy in combating AMR, but one which requires more research to guide intervention prioritization.^6^ The potential for influenza vaccines to reduce antibiotic prescribing has been determined in only one study from Ontario, Canada, in which an association between a 64% reduction in antibiotic prescriptions and roll-out of a universal influenza immunization programme was demonstrated.^7^ The impact of influenza vaccine roll-out on antibiotic usage in Africa is currently unknown.

In the absence of direct trial data, we combined data from a range of sources to predict the potential number of antibiotic prescriptions that could be directly avoided by influenza vaccine roll-out in various African populations, taking into account variability in healthcare (and therefore antibiotic) availability and vaccine coverage. These estimates should stimulate further discussion and research on the wider benefits of influenza vaccine roll-out in African countries with currently low influenza vaccine coverage, high influenza burden, high level of antibiotic use and rising levels of AMR.

## Methods

### Data on influenza incidence

There is limited information on many aspects required to comprehensively estimate the impact of influenza vaccination on antibiotic prescribing across Africa. Hence, we included only the number of (i) appropriate antibiotic prescriptions following SARI and (ii) inappropriate antibiotic prescriptions following influenza-related ILI in example settings. We identified studies that provided robust estimates of influenza-related ILI or SARI in different high-risk groups from a number of African countries, either via attack rates in placebo recipients enrolled in randomized clinical trials or epidemiological studies and systematic reviews [Table S1 (see Supplementary data—Part I, available as Supplementary data at *JAC* Online)]. We did not include the indirect impact of vaccination on secondary influenza cases owing to a lack of data on influenza transmission dynamics from African settings.

**Table 1.** Estimated number of antibiotic prescriptions that could be averted per year by the introduction of an influenza vaccine into specific high-risk groups in Africa, where we could find sufficient data

Population	Setting	ILI	SARI	Number of prescriptions averted per year, mean (range)		
				total	per 100 000 population	per 10 000 vaccinations
≥65 years old	South Africa	x		11 153	399	133
	Ghana	x	x	140 (125–157)	15 (13–17)	5 (4.5–5.6)
<5 years old	Kenya	x		9425 (6492–13 655)	135 (93–195)	44.9 (30.9–65.1)
	Ghana	x	x	8456 (8233–8691)	210 (205–216)	70.1 (68.2–72)
2–5 years old	Senegal	x		13 772	945	315.0
<6 months old	South Africa	x		1094	189	63.0
	Mali	x		505	147	49.0
	Kenya	x	x	894 (254–3434)	128 (36–491)	42.6 (12.1–163.7)
Pregnant	South Africa	x		1661	189	63.0
	Mali	x		565	100	33.3
<5 years old	Africa^a^		x	24 (12–49)^a^	13 (7–26)	4.4 (2.2–8.7)
	Africa^a^		x	25 (14–47)^a^	14 (7–25)	4.5 (2.4–8.3)

A cross (‘x’) indicates where estimates came from: ILI, SARI or both. The range given is a 95% CI except for Kenyan data for which it is minimum–maximum. See Table S1 for sources of incidence data for each example. Vaccine effectiveness was assumed to be 50%, vaccine coverage 30% and antibiotic availability 50%. Estimates for other coverage and antibiotic availability can be found in Supplementary data – Part II.

a Note that the values for the estimates for the African setting total are in thousands of prescriptions.

### Calculating antibiotic use

We split antibiotic use into two components: (i) likelihood that someone with an ILI or SARI would be prescribed antibiotics; and (ii) likely provision of healthcare and antibiotics in a setting. These were multiplied to give a level of antibiotic prescribing.

For (i) we assumed that SARI cases would usually fulfil criteria in clinical guidelines for prescribing antibiotics (e.g. WHO integrated management of childhood illness) and therefore that, if available, 100% would be prescribed antibiotics. The available literature suggests that the proportion with ILI that receive an (inappropriate) antibiotic is higher in LMICs than in high-income settings (see Supplementary data—Part I), hence we assumed in our calculations that 70% of influenza-related ILI would inappropriately be prescribed antibiotics.

We assumed that coverage of healthcare provision and antibiotic availability was 50%. Thus, even if 100% of SARI patients would ordinarily be given antibiotics, only 50% of them would receive antibiotics. The aim of this parameter was to reflect health system failings in LMIC settings where antibiotics may not always be available, despite prescription, or where SARI-related deaths occur outside a healthcare setting.

### Population size estimates

Data from the World Bank for 2015 were used to generate population size estimates (see Supplementary data—Part I).

### Vaccine characteristics and coverage

We assumed vaccine effectiveness was 50% based on various international estimates.^8^ We considered a low vaccine coverage of 30%. In Supplementary data—Part II we provide estimates for higher healthcare provision and antibiotic availability (80%) and 90% vaccine coverage. The high vaccine coverage figure was based on studies in The Gambia, where uptake of infant immunizations reaches >90% in many cases.^9^

## Results

The overall estimates for the impact of an influenza vaccine programme targeting key high-risk groups are shown in Table 1. With low vaccine coverage (30%) and antibiotic availability at 50%, the number of prescriptions that could be averted by targeting each risk group is between 15 and 945 per 100 000 population per year. Of the populations considered, the lowest estimates come from targeting those >65 years old in Ghana, the highest from targeting adults >65 years old in South Africa or children 2–5 years old in Senegal. In a corresponding measure, 5–315 antibiotic prescriptions could be averted per 10 000 vaccinations.

Two studies provided estimates for SARI incidence only in children <5 years old across Africa.^4^^,^^10^ Using these, we estimated that just avoiding appropriate antibiotic use for these most serious cases with the introduction of influenza vaccine at 30% coverage could prevent at least 24 000 antibiotic prescriptions per year [13 (95% CI 7–26) per 100 000 population per year].

## Discussion

We aimed to estimate the impact of influenza vaccines on antibiotic use in Africa, using the current limited data available. Our conservative direct impact estimates suggest that a large number of antibiotic prescriptions could be averted across Africa each year, even with low coverage of an influenza vaccine.

Our estimates were limited by a lack of data. More data are needed on both influenza and secondary bacterial infection incidence, as well as antibiotic exposure levels (by age) to allow calculation of ‘influenza-attributable prescribing’.^11^ In addition, determining influenza vaccine impact would involve modelling vaccine campaign timing (with varying influenza seasonality across Africa) and variation in coverage in different risk populations. Vaccine efficacy may also vary in different risk populations (e.g. due to immunosenescence), as well as due to seasonality and influenza antigenic drift. Moreover, high HIV prevalence in certain settings, alongside substantial variation in access to healthcare (and hence antibiotic prescribing) could make estimates highly setting-specific. We included an antibiotic ‘availability’ parameter, but to our knowledge, there are no studies that explore the relative ease of antibiotic accessibility across Africa (e.g. impact of unsanctioned providers, health system quality or rural/economic setting) or health-seeking behaviour differences.

Our evaluation is an underestimate, not only as we likely use conservative vaccine coverage (30%) and antibiotic availability (50%) values, but as we do not include the indirect impact of vaccination on secondary cases of influenza. Reduction in influenza transmission in the community by vaccinating high-risk groups may significantly enhance the impact observed. A recent modelling study of the German population suggested that 4–7× as many influenza cases are prevented among non-vaccinated individuals as among vaccinees.^12^ Owing to a lack of data, our estimates also only considered the number of ILI or SARI cases averted by the vaccine. Only a minority of risk groups (e.g. **≥**65 years old in Ghana) had data on both ILI and SARI incidence (Table 1). Hence our estimates are an underestimate of even the combined direct impact of vaccination.

Several agencies (e.g. GAVI) are now calling for the use of vaccines to help in the prevention of AMR.^11^ However, as in our work here, although the impact on antibiotic prescribing can be estimated, the jump to impact on AMR is challenging to make.^13^ Without this link, the likely dramatic impact of influenza vaccine on antibiotic usage and subsequent AMR levels in Africa cannot be estimated.

The estimates we make here should be expanded as more data on influenza and antibiotic use become available. Importantly, future trials in LMICs should consider linking outcomes across public health measures; influenza vaccine trials could be designed to capture impact on antibiotic usage in addition to preventing influenza infections.

Influenza vaccines could have a dramatic impact on morbidity and mortality in Africa. The reasons for the lack of influenza vaccine programmes across the continent are multifactorial, including health economic ones. Yet policy decisions are often made by considering prevention of influenza infections as the sole beneficial outcome. Although public health interventions such as vaccination are costly, as highlighted by our estimates, the wider benefits may be substantial and, with increasing evidence, should be included as key considerations.

## Supplementary Material

Supplementary Data
