# Supplementary material for: Potential impact of influenza vaccine roll-out on antibiotic use in Africa
Source: J Antimicrob Chemother. 2018 May 9;73(8):2197–200. doi: 10.1093/jac/dky172 (PMC6054263; doi:10.1093/jac/dky172)
Supplement: Supplementary Data [file dky172_suppl_data.zip › Supplementary_Data_I.docx]

**Supplementary data – Part I**

**Additional methods**

***Inappropriate antibiotic prescribing***

We assumed that not everyone with influenza-associated respiratory illness would receive an antibiotic. The available literature suggests that the proportion with ILI that receive an (inappropriate) antibiotic is higher in LMICs than in high income settings. In the United States, one study found that 21.65% of influenza patients received antibiotics, with 79% being inappropriate prescriptions ^1^. In Sri Lanka, 83.7% of all influenza-associated ILI received antibiotics ^2^. Studies in Thailand and Turkey have suggested antibiotic prescribing for any ILI is 82% and 100% respectively ^3, 4^. In Zanzibar, 74% (n=500) of 677 children aged 2-5yrs with acute uncomplicated febrile illness received antibiotics, although only 22% were considered to have an infection that required antibiotics ^5^. From this evidence base, we assumed in our calculations that 70% of ILI with influenza would inappropriately be prescribed antibiotics.

***Population size estimates***

Data from the World Bank for 2015 was used to generate population size estimates ^6^. The population aged ≥65 was calculated using the “Population ages 65 and above (% of total)” multiplied by the population total (“Population, total”). The population <5 years old was the sum of the male and female population aged 00-04. The number of infants <6 months old was taken to be half the population aged 0. The number of pregnant women was taken to be 2/3 (9/12month) of the total number of births per year, calculated using the birth rate (“Birth rate, crude (per 1,000 people)”) multiplied by the total population size.

***Calculation of vaccine impact***

Method:

1. Mean and ranges of Incidence of influenza associated ILI, and SARI, was gathered from available literature for individual risk groups and country settings. Where information was available on HIV status, only data from HIV uninfected individuals was included.
2. These were standardised to an incidence rate per year
3. The population size for each risk group was gathered from the World Bank
4. The incidence estimates (mean and range where available) were multiplied by population size to give the estimated number with ILI or SARI in each risk group, in each country per year.
5. The estimated number of antibiotic prescriptions was then calculated at the “antibiotic availability” level required.
6. The impact of reducing this number by a 50% efficacious vaccine, at varying coverage levels, was then calculated.

See Supplementary data – Part II (Excel spreadsheets) for all data and direct calculations. Included in this .xls is the summary data, the calculations for the model and a final page with a gathering of data for presentation in the manuscript. The variable parameters (vaccine efficacy, vaccine coverage, antibiotic availability, prescribing levels) can be changed in the sheet “Calculations” for further investigation if desired.

**Table S1.** Sources of data included in estimates

| **Population** | **Setting** | **ILI** | **SARI** | **Reference** | |
| --- | --- | --- | --- | --- | --- |
| **≥ 65 yo** | South Africa | x |  | ^7^ | De Villiers PJT, Steele AD, Hiemstra LA, et al. Efficacy and safety of a live attenuated influenza vaccine in adults 60 years of age and older. Vaccine **2009** |
|  | Ghana | x | x | ^8^ | Ntiri MP, Duque J, McMorrow ML, et al. Incidence of medically attended influenza among residents of Shai-Osudoku and Ningo-Prampram Districts, Ghana, May 2013 – April 2015. BMC Infectious Diseases **2016**; 16: 757. |
| **< 5 yo** | Kenya | x |  | ^9^ | Emukule GO, Khagayi S, McMorrow ML, Ochola R, Otieno N, et al. The Burden of Influenza and RSV among Inpatients and Outpatients in Rural Western Kenya, 2009–2012. PLoS ONE **2014** 9(8): e105543 |
|  | Ghana | x | x | ^8^ | Ntiri MP, Duque J, McMorrow ML, et al. Incidence of medically attended influenza among residents of Shai-Osudoku and Ningo-Prampram Districts, Ghana, May 2013 – April 2015. BMC Infectious Diseases **2016**; 16: 757. |
| **(2-5yo)** | Senegal | x |  | ^10^ | Victor JC, Lewis KDC, Diallo A, et al. Efficacy of a Russian-backbone live attenuated influenza vaccine among young children in Bangladesh: a randomised, double-blind, placebo-controlled trial. The Lancet Global Health **2016**; 4: e946-e54. |
| **< 6 mo** | South Africa | x |  | ^11^ | Madhi SA, Cutland CL, Kuwanda L, et al. Influenza Vaccination of Pregnant Women and Protection of Their Infants. New England Journal of Medicine **2014**; 371: 918-31. |
|  | Mali | x |  | ^12^ | Tapia MD, Sow SO, Tamboura B, et al. Maternal immunisation with trivalent inactivated influenza vaccine for prevention of influenza in infants in Mali: a prospective, active-controlled, observer-blind, randomised phase 4 trial. The Lancet Infectious Diseases **2016**; 16: 1026-35. |
|  | Kenya | x | x | ^9^ | Emukule GO, Khagayi S, McMorrow ML, Ochola R, Otieno N, et al. The Burden of Influenza and RSV among Inpatients and Outpatients in Rural Western Kenya, 2009–2012. PLoS ONE **2014** 9(8): e105543 |
| **Pregnant** | South Africa | x |  | ^11^ | Madhi SA, Cutland CL, Kuwanda L, et al. Influenza Vaccination of Pregnant Women and Protection of Their Infants. New England Journal of Medicine **2014**; 371: 918-31. |
|  | Mali | x |  | ^12^ | Tapia MD, Sow SO, Tamboura B, et al. Maternal immunisation with trivalent inactivated influenza vaccine for prevention of influenza in infants in Mali: a prospective, active-controlled, observer-blind, randomised phase 4 trial. The Lancet Infectious Diseases **2016**; 16: 1026-35. |
| **< 5 yo** | Africa |  | x | ^13^ | Lafond KE, Nair H, Rasooly MH, et al. Global Role and Burden of Influenza in Pediatric Respiratory Hospitalizations, 1982–2012: A Systematic Analysis. PLoS Medicine **2016**; 13: 1-19. |
|  | Africa |  | x | ^14^ | Nair H, Brooks WA, Katz M, et al. Global burden of respiratory infections due to seasonal influenza in young children: A systematic review and meta-analysis. The Lancet **2011**; 378: 1917-30. |

ILI = influenza-like illness, SARI = severe acute respiratory infection, yo = years old.

**References**

1. Misurski DA, Lipson DA, Changolkar AK. Inappropriate antibiotic prescribing in managed care subjects with influenza. *Am J Manag Care* 2011; **17**: 601-8.

2. Tillekeratne LG, Bodinayake CK, Nagahawatte A et al. Use of Rapid Influenza Testing to Reduce Antibiotic Prescriptions Among Outpatients with Influenza-Like Illness in Southern Sri Lanka. *Am J Trop Med Hyg* 2015; **93**: 1031-7.

3. Bhavnani D, Phatinawin L, Chantra S et al. The influence of rapid influenza diagnostic testing on antibiotic prescribing patterns in rural Thailand. *Int J Infect Dis* 2007; **11**: 355-9.

4. Ozkaya E, Cambaz N, Coskun Y et al. The effect of rapid diagnostic testing for influenza on the reduction of antibiotic use in paediatric emergency department. *Acta Paediatr* 2009; **98**: 1589-92.

5. Elfving K, Shakely D, Andersson M et al. Acute Uncomplicated Febrile Illness in Children Aged 2-59 months in Zanzibar - Aetiologies, Antibiotic Treatment and Outcome. *PLoS One* 2016; **11**: e0146054.

6. The World Bank. DataBank: Population estiamtes and projections. 2017.

7. De Villiers PJT, Steele AD, Hiemstra LA et al. Efficacy and safety of a live attenuated influenza vaccine in adults 60 years of age and older. *Vaccine* 2009.

8. Ntiri MP, Duque J, McMorrow ML et al. Incidence of medically attended influenza among residents of Shai-Osudoku and Ningo-Prampram Districts, Ghana, May 2013 – April 2015. *BMC Infectious Diseases* 2016; **16**: 757.

9. Emukule GO, Khagayi S, McMorrow ML et al. The burden of influenza and RSV among inpatients and outpatients in rural western Kenya, 2009-2012. *PLoS One* 2014; **9**: e105543.

10. Victor JC, Lewis KDC, Diallo A et al. Efficacy of a Russian-backbone live attenuated influenza vaccine among young children in Bangladesh: a randomised, double-blind, placebo-controlled trial. *The Lancet Global Health* 2016; **4**: e946-e54.

11. Madhi SA, Cutland CL, Kuwanda L et al. Influenza Vaccination of Pregnant Women and Protection of Their Infants. *New England Journal of Medicine* 2014; **371**: 918-31.

12. Tapia MD, Sow SO, Tamboura B et al. Maternal immunisation with trivalent inactivated influenza vaccine for prevention of influenza in infants in Mali: a prospective, active-controlled, observer-blind, randomised phase 4 trial. *The Lancet Infectious Diseases* 2016; **16**: 1026-35.

13. Lafond KE, Nair H, Rasooly MH et al. Global Role and Burden of Influenza in Pediatric Respiratory Hospitalizations, 1982–2012: A Systematic Analysis. *PLoS Medicine* 2016; **13**: 1-19.

14. Nair H, Brooks WA, Katz M et al. Global burden of respiratory infections due to seasonal influenza in young children: A systematic review and meta-analysis. *The Lancet* 2011; **378**: 1917-30.
